# Two new species of the genus *Anufrievia* Dworakowska (Hemiptera, Cicadellidae, Typhlocybinae) from karst region of southwest China

**DOI:** 10.3897/BDJ.10.e77511

**Published:** 2022-03-01

**Authors:** Zhouwei Yuan, Ni Zhang, Yuehua Song

**Affiliations:** 1 School of Karst Science, Guizhou Normal University / State Key Laboratory Cultivation Base for Guizhou Karst Mountain Ecology Environment of China, Guiyang, 550001, China, Guiyang, China School of Karst Science, Guizhou Normal University / State Key Laboratory Cultivation Base for Guizhou Karst Mountain Ecology Environment of China, Guiyang, 550001, China Guiyang China

**Keywords:** Cicadellidae, Erythroneurini, taxonomy, morphology, new species

## Abstract

**Background:**

The leafhopper genus *Anufrievia* was established by Dworakowska with *A.rolikae* Dworakowska as its type species. They are widely distributed in China, North Korea, South Korea, Japan, Nepal, India, Indonesia, Thailand and Vietnam.

**New information:**

Two new species, *A.ventricosa* **sp. nov**. and *A.unianxialis* **sp. nov**., found in the karst area of Guanling and Shibing City, Guizhou Province, China are described and illustrated. The key to the identification of the specie of this genus is given.

## Introduction

The leafhopper genus *Anufrievia* belongs to the tribe Erythroneurini of Typhlocybinae and previously contained 35 species, including 27 species in China (Cao et al. 2018, Tan et al. 2021). In this study, two new species from the karst area of Guizhou Province, China are described and illustrated. The key to the identification of the specie of this genus is given.

***Anufrievia* Dworakowska, 1970**

*Anufrievia* Dworakowska 1970: 761; Chiang and Knight 1990: 195

Type species: *Anufrieviarolikae* Dworakowska 1970

Main characteristics of genus *Anufrievia* were described as follows. Body brown or yellow. Head slightly narrower than pronotum. Length of crown obviously shorter than inter-ocular width. Vertex moderately produced medially; anterior margin usually with small paired dark spots, sometimes absent. Pronotum broad, with anterior margin convex and posterior margin slightly concave. Scutellum with dark lateral triangles. Face without black spots anterodorsad of antennal pits. Anteclypeus pale, concolorous with rest of face or brown or black. Forewing with 4^th^ apical cell small, not reaching apex of forewing, 2nd apical cell nearly rectangular and 1st apical cell broad. Hind-wing apex broadly rounded. Hind-wings' venation follows typical schemes for Erythroneurini taxa. Abdominal apodemes short, extended dorsomesad.

Male pygofer lobe with hind margin sleeked or truncated slightly, cephalo-ventral angle usually with macrosetae, sometimes absent and scattered with a few fine setae in outer lateral surface. Pygofer dorsal appendage movably articulated to pygofer lobe with ventral appendage absent. Subgenital plate broad basally, tapering to middle, subparallel-sided from middle to apex and rounded apically; with some macrosetae in mid-ventral part; row of stout setae along upper margin almost from subbase to apex. Style with shape of apex various, bifid, foot-shaped or otherwise modified. Aedeagus with aedeagal shaft tubular; gonopore sub-basal to subapical on ventral surface; with or without well-developed pre-atrial processes, dorsal apodeme well developed. Connective lateral arms long Y- or V-shaped.

## Materials and methods

The specimen was collected by the sweeping-net method. Morphological terminology used follows Dietrich (2005) and Dworakowska (1993). An Olympus SZX16 dissecting microscope was used for observing and an Olympus BX53 stereomicroscope for drawing. A KEYENCE VHX-5000 digital microscope was used for taking habitus photos. Body measurements are measured from the apex of the vertex to the tip of the forewing. Male specimens were selected under a stereoscope, the entire abdomen of the specimens was removed and soaked in 10% sodium hydroxide (NaOH) solution or 10% potassium hydroxide (KOH) solution for 15-20 hours. After that, the abdomen was rinsed with clean water, drained of the excess water with qualitative filter paper and transferred to a clean glass dripping with glycerine. All specimens examined were deposited in the collection of the School of Karst Science, Guizhou Normal University/State Key Laboratory Cultivation Base for Guizhou Karst Mountain Ecology Environment of China, Guiyang, China.

## Taxon treatments

### Anufrievia ventricosa sp. n.

E627C28E-DB7D-5662-936A-33DCC5B9D494

D493BC2C-A404-4FA4-855C-F86A785AF6AF

#### Materials

- **Type status:** Holotype. **Occurrence:** individualCount: "1"; sex: male; lifeStage: adult; **Taxon:** scientificName: *Anufrieviaventricosa*; order: Hemiptera; family: Cicadellidae; genus: Anufrievia; specificEpithet: *ventricosa*; **Location:** country: China; stateProvince: Guizhou; county: Guanling; locationRemarks: "Guizhou, Guanling, 27. 9. 2020, coll. Zhouwei Yuan and Xiao Yang"; **Record Level:** collectionCode: Insects; basisOfRecord: Preserved Specimen

#### Description

**Figure 1.**
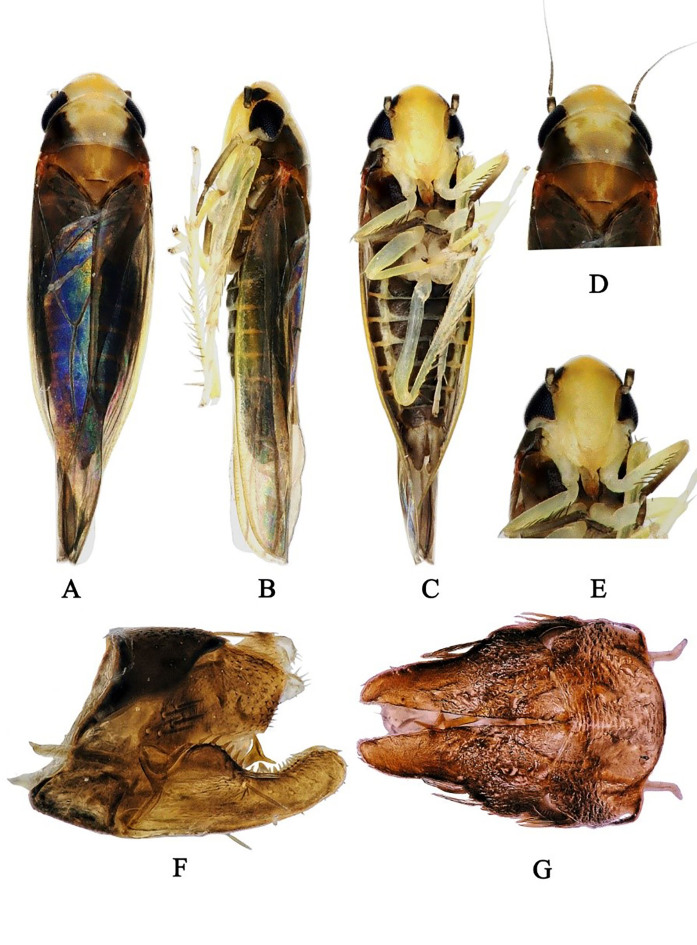
*Anufrieviaventricosa* **sp. nov**. **A** Habitus, dorsal view; **B** Habitus, lateral view; **C** Habitus, ventral view; **D** Head and thorax, dorsal view; **E** Face; **F** Genital capsule, lateral view; **G** Genital capsule, ventral view.

**Figure 2.**
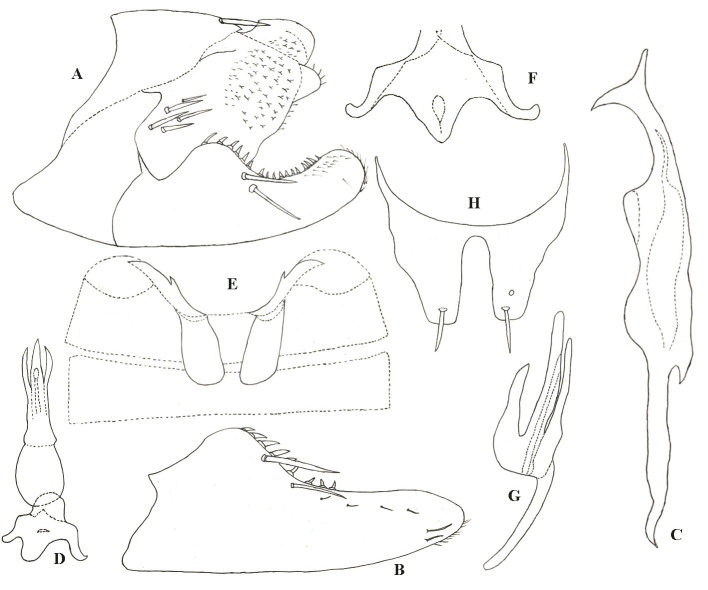
*Anufrieviaventricosa* **sp. nov**. **A** Pygofer lobe; **B** Subgenital plate; **C** Style; **D** Aedeagus, ventral view; **E** Abdominal apodemes; **F** Connective; **G** Aedeagus, lateral view; **H** Part of pygofer lobe, dorsal view.

Body brownish-black. Head milky yellow (Fig. 1A and D). Eyes pitch black. Face milky yellow, anteclypeus little darker than fronteclypeus (Fig. 1C and E). Pronotum light yellow with two sides black (Fig. 1A and D). Scutellum yellowish-brown, with dark basal triangles (Fig. 1A and D). Forewing brownish (Fig. 1A and C). Abdominal apodemes broad, extended to 4^th^ sternite (Fig. 2C).

#### Diagnosis

**Male genitalia**

Pygofer lobe broad, with five macroseta at cephalo-ventral angle of lobe and one macroseta at junction area with anal tube (Fig. 2A). Pygofer dorsal appendage broad at base, tapering towards apex. Subgenital plate slightly concave near middle area, with two macrosetae on outer surface (Fig. 2B). Style with two points at apex; pre-apical lobe small (Fig. 2C). Aedeagus with shaft almost straight and flat in lateral view, pair of long processes arising from base of shaft, surpassing gonopore; gonopore reaching 3/4 height of aedeagal shaft, on ventral surface; dorsal apodeme well developed (Fig. 2D and G). Connective Y-shaped, two lateral arms long and central lobe large; stem well developed (Fig. 2F).

**Measurement**: Male length (including wing) 2.9 mm.

#### Etymology

The specific epithet is derived from the Latin word “*ventricosus*”, referring to the connective central lobe well developed.

#### Taxon discussion

This species is similar to *A.confluensa* Tan, Jiang & Song, 2021 with similar shape of style and aedeagus, but can be distinguished by the five macrosetae at cephalo-ventral angle of lobe and one macroseta at junction area with anal tube; the aedeagus with pair of long processes arising from base of shaft and the connective central lobe well developed.

### Anufrievia unianxialis sp. n.

367FAFF4-859D-5069-8F9D-83989425EA8D

840D1AE6-EB79-4702-A8C3-092446B96CD4

#### Materials

- **Type status:** Holotype. **Occurrence:** individualCount: 1; sex: male; lifeStage: adult; **Taxon:** scientificName: *Anufrieviaunianxialis*; order: Hemiptera; family: Cicadellidae; genus: Anufrievia; specificEpithet: *unianxialis*; **Location:** country: China; stateProvince: Guizhou; county: Shibing; locationRemarks: Guizhou, Shibing, 27. 6. 2021, coll. Zhouwei Yuan and Jiang Jia; **Record Level:** collectionCode: Insects; basisOfRecord: Preserved Specimen

#### Description

**Figure 3.**
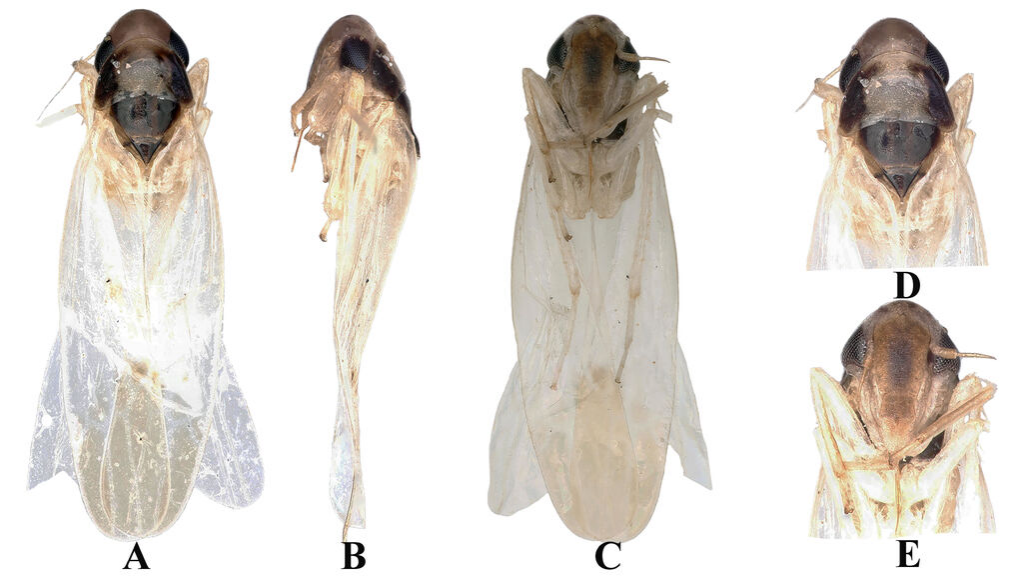
*Anufrieviaunianxialis* sp. nov. **A** Habitus, dorsal view; **B** Habitus, lateral view; **C** Habitus, ventral view; **D** Head and thorax, dorsal view; **E** Face.

**Figure 4.**
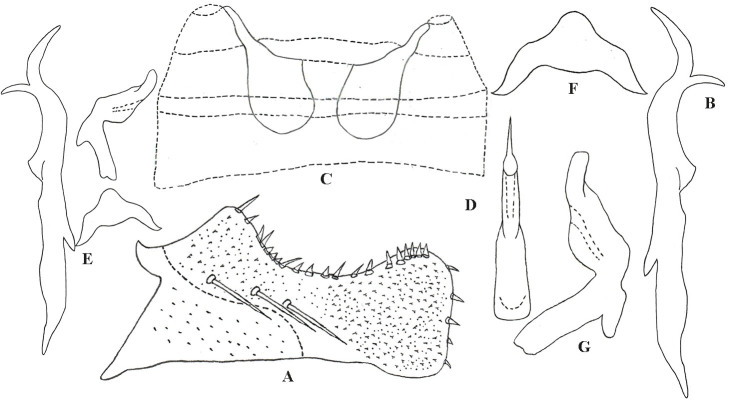
*Anufrieviaunianxialis* sp. nov. **A** Subgenital plate; **B** Style; **C** Abdominal apodemes; **D** Aedeagus, ventral view; **E** Aedeagus, style and connective; **F** Connective; **G** Aedeagus, ventrolateral view.

Body brownish-yellow (Fig. 3A). Head brownish-black (Fig. 3A and D). Eyes black. Face brownish, anteclypeus brownish and frontoclypeus centrally dark brown with brownish lateral margins (Fig. 3C and E). Pronotum brownish two sides black (Fig. 3A and D). Scutellum black in whole (Fig. 3A and D). Forewing faint brown (Fig. 3A and C). Abdominal apodemes broad, extended to 4^th^ sternite (Fig. 4C).

#### Diagnosis

**Male genitalia**

Subgenital plate slightly concave near middle area, with three macrosetae on outer surface (Fig. 4A). Style with two points at apex; pre-apical lobe obvious (Fig. 4B). Aedeagal shaft almost straight and flat in lateral view, without any process; gonopore large, reaching 1/2 height of aedeagal shaft; dorsal apodeme small and pre-atrium well developed, as long as shaft (Fig. 4D and G). Connective M-shaped, two lateral arms long, central lobe small (Fig. 4F).

**Measurement**: Male length (including wing) 2.7 mm.

#### Etymology

The new species is named from the Latin word “*unianxialis*”, referring to the aedeagal shaft without serrated marginal lamellae apically.

#### Taxon discussion

This species is similar to *A.crispata* Tan, Jiang & Song, 2021, but can be distinguished by the aedeagal shaft without serrated marginal lamellae; without pair of small processes curved mesally under the gonopore and the connective with distinct central lobe.

## Identification Keys

### Key to males of *Anufrievia* from China (modified from Tan *et al*. 2021)

**Table:** 

1	Pygofer dorsal appendage not bifurcate at apex	2
–	Pygofer dorsal appendage bifurcate at apex	15
2	Aedeagus with large dorsal apodeme	4
–	Aedeagus with small dorsal apodeme	3
3	Aedeagal shaft with serrated marginal lamellae apically	*A.crispata* Tan, Jiang & Song
–	Aedeagal shaft without serrated marginal lamellae apically (Fig. 4D)	*A.unianxialis* **sp. nov.**
4	Pre-atrial process not reaching gonopore	5
–	Pre-atrial process reaching or surpassing gonopore	9
5	Style without distinct apical and subapical teeth	6
–	Style with distinct apical and subapical teeth	7
6	Style with apex slim	*A.symmetrica* Cao & Zhang
–	Style with apex triangular	*A.triangulata* Cao & Zhang
7	Pre-atrial process almost rectangular in ventral view, apex broad	*A.quadrata* Cao & Zhang
–	Pre-atrial process narrowing apically, apex pointed	8
8	Style with subapical tooth equal in length to apical tooth	*A.adaucta* Cao & Zhang
–	Style with subapical tooth shorter than apical tooth	*A.sphenoides* Yang & Zhang
9	Aedeagal shaft with pair of apical processes	10
–	Aedeagal shaft without any apical processes	12
10	Aedeagal apical processes arched medially in ventral view	A. arcuata Yang & Zhang
–	Aedeagal apical processes slightly curved in ventral view	11
11	Aedeagal shaft with base slim, slightly wider than apex	*A.zelta* Dworakowska
–	Aedeagal shaft with base broad, much wider than apex	13
12	Connective central lobe well developed (Fig. 2F)	*Anufrieviaventricosa* **sp. nov.**
–	Connective central lobe absent	*A.confluensa* Tan, Jiang & Song
13	Aedeagal shaft constricted sub-basally	*A.jinghongensis* Cao & Zhang
–	Aedeagal shaft not constricted sub-basally	14
14	Style with apical tooth extremely small, aedeagal shaft straight	*A.subdentata* Yang & Zhang
–	Style with apical tooth relatively long, aedeagal shaft curved dorsad	*A.ciconia* Dworakowska
15	Aedeagal shaft with processes near middle	*A.triprocessa* Yang & Zhang
–	Aedeagal shaft without process near middle	16
16	Apex of style serrated at middle	17
–	Apex of style smooth at middle	20
17	Upper tooth of pygofer dorsal appendage much shorter than lower one	*A.bauhinicola* Dworakowska & Viraktamath
–	Upper tooth of pygofer dorsal appendage subequal to or longer than lower one	18
18	Upper tooth of pygofer dorsal appendage longer than lower one	*A.expansa* Cao & Zhang
–	Upper tooth of pygofer dorsal appendage almost as long as lower one	19
19	Apex of pre-atrial process rounded, with one side serrated	*A.plana* Yang & Zhang
–	Apex of pre-atrial process truncate, with both sides smooth	*A.curva* Yang & Zhang
20	Ventral margin of aedeagal shaft protruded subapically in lateral view	21
–	Ventral margin of aedeagal shaft straight subapically, in lateral view	23
21	Apical tooth of style almost equal to subapical tooth	*A.liubanus* Yang & Zhang
–	Apical tooth of style greatly shorter than subapical tooth	22
22	Aedeagal shaft processes relatively long, gonopore central	*A.parisakazu* Cao & Zhang
–	Aedeagal shaft processes relatively short, gonopore subapical	*A.akazu* Matsumura
23	Apex of pre-atrial process serrated laterally	*A.fusina* Yang & Zhang
–	Apex of pre-atrial process smooth	24
24	Pre-atrial process rudimentary, as long as 1/5 of aedeagal shaft	*A.badjawae* Dworakowska
–	Pre-atrial process much longer than 1/5 of aedeagal shaft	25
25	Aedeagal shaft curved dorsad	*A.falcata* Yang & Zhang
–	Aedeagal shaft straight	26
26	Apex of style slender	*A.qinlingensis* Yang & Zhang
–	Apex of style foot-like	27
27	Aedeagal shaft with processes arising from subapex	28
–	Aedeagal shaft with processes arising from apex	29
28	Apex of aedeagal shaft expanded	*A.forcipiformis* Yang & Zhang
–	Apex of aedeagal shaft narrow	*A.subapicifixa* Yang & Zhang
29	Aedeagal shaft processes bent at right angle in ventral view	*A.rolikae* Dworakowska
–	Aedeagal shaft processes straight or slightly curved in ventral view	30
30	Style without distinct apical and subapical teeth	*A.sufflata* Yang & Zhang
–	Style with distinct apical and subapical teeth	31
31	Gonopore subapical	*A.wolongensis* Yang & Zhang
–	Gonopore central	*A.maculosa* Dworakowska

## Supplementary Material

XML Treatment for Anufrievia ventricosa

XML Treatment for Anufrievia unianxialis
